# The Influence of Oxidative Stress on Thyroid Diseases

**DOI:** 10.3390/antiox10091442

**Published:** 2021-09-10

**Authors:** Joanna Kochman, Karolina Jakubczyk, Piotr Bargiel, Katarzyna Janda-Milczarek

**Affiliations:** 1 Department of Human Nutrition and Metabolomics, Pomeranian Medical University, 24 Broniewskiego Street, 71-460 Szczecin, Poland; kochmaan@gmail.com (J.K.); katarzyna.janda-milczarek@pum.edu.pl (K.J.-M.); 2 Clinic of Plastic, Endocrine and General Surgery, Pomeranian Medical University, 2 Siedlecka Street, 72-010 Police, Poland; bergiel87@gmail.com

**Keywords:** oxidative stress, ROS, thyroid diseases, antioxidants

## Abstract

Thyroid diseases, including neoplasms, autoimmune diseases and thyroid dysfunctions, are becoming a serious social problem with rapidly increasing prevalence. The latter is increasingly linked to oxidative stress. There are many methods for determining the biomarkers of oxidative stress, making it possible to evaluate the oxidative profile in patients with thyroid diseases compared to the healthy population. This opens up a new perspective for investigating the role of elevated parameters of oxidative stress and damage in people with thyroid diseases, especially of neoplastic nature. An imbalance between oxidants and antioxidants is observed at different stages and in different types of thyroid diseases. The organ, which is part of the endocrine system, uses free radicals (reactive oxygen species, ROS) to produce hormones. Thyroid cells release enzymes that catalyse ROS generation; therefore, a key role is played by the internal defence system and non-enzymatic antioxidants that counteract excess ROS not utilised to produce thyroid hormones, acting as a buffer to neutralise free radicals and ensure whole-body homeostasis. An excess of free radicals causes structural cell damage, undermining genomic stability. Looking at the negative effects of ROS accumulation, oxidative stress appears to be implicated in both the initiation and progression of carcinogenesis. The aim of this review is to investigate the oxidation background of thyroid diseases and to summarise the links between redox imbalance and thyroid dysfunction and disease.

## 1. Introduction

Reactive oxygen species (ROS) are molecules capable of independent existence, which contain an oxygen atom and unpaired electrons [1]. ROS arise mainly as by-products in a series of bioenergetic processes of ATP synthesis in mitochondrial respiratory chains [2,3]. Inflammatory processes are an additional source of ROS [1,4]. The most common reactive oxygen species include radicals derived from the electron reduction of molecular oxygen–superoxide anion (O_2_^•−^), hydrogen peroxide (H_2_O_2_) and the more reactive hydroxyl radical (HO^•^), released in reactions involving metal ions [5].

The body’s antioxidant defence against the negative effects of ROS works across a number of different platforms. It involves preventing the formation of radicals, scavenging them and repairing ROS-induced damage. The leading role in the body’s defence system is played by antioxidant enzymes, breaking down ROS molecules and thus protecting cells from excessive exposure to ROS [6,7,8]. The repair system of ROS-induced damage partly relies on autophagy and apoptosis processes, eliminating damaged cells [9,10,11]. In spite of the range of internal mechanisms of enzymatic regulation, the antioxidant defence system should also be supported by non-enzymatic mechanisms. The latter include the action of molecules with powerful antioxidant properties, notably including glutathione, coenzyme Q10, as well as exogenous substances—polyphenolic compounds, ascorbic acid, retinol, β-carotene and tocopherol. Exogenous substances with confirmed antioxidant properties reinforce the body antioxidant defence, increasing total antioxidant capacity [7,12,13,14].

Oxidative stress is an effect of redox imbalance between reactive oxygen species and antioxidant defence [9,15]. It may be caused both by the excessive production of ROS and by an inefficient antioxidant system, resulting in molecular damage [16]. Additionally, ROS generation in different subcellular compartments likely involves a positive feedback mechanism, creating a vicious circle of pathological conditions related to oxidative stress [17,18,19]. Redox homeostasis requires an equilibrium of ROS production and scavenging [20]. Even though the concept of oxidative stress was introduced in the 1980s, its definition and scope of research have been continually elaborated and expanded [6].

Thyroid diseases are a common health problem worldwide, especially among women. The occurrence of subclinical thyroid disorders, which often remain undiagnosed, is also significant [21,22,23,24]. Thyroid diseases are increasingly linked to oxidative stress [25,26,27,28]. It has been shown that thyroid dysfunction can co-occur with metabolic disorders, including obesity [29,30,31]. Obesity is a metabolic disease involving mitochondrial dysfunction and chronic oxidative stress, as in several metabolic disorders [32,33,34,35,36,37,38]. Since the incidence of thyroid diseases is increased in individuals with increased body weight, the related substrate of metabolic disorders and thyroid dysfunction seems relevant [30,31,39]. However, current reports do not distinguish between the causes and consequences of metabolic abnormalities, so there is a need to develop research on the pathogenesis of thyroid disorders.

## 2. Physiological Redox Signalling and the Role of ROS in Thyroid Function

Signalling functions in immune responses are initiated when molecular oxygen is oxidised to the reactive superoxide anion radical by the NADPH oxidase (NOX) complex, itself an additional source of ROS [4]. Subsequently, the superoxide is converted by superoxide dismutase (SOD) to H_2_O_2_. Hydrogen peroxide is associated with a signalling function regulating cellular processes, due to its capacity to reversibly modify cysteine residues [20]. The process alters redox signalling [17]. Accumulation of excessive concentrations of H_2_O_2_ activates thiolate anion (Cys-S-) oxidation pathways. This is an irreversible process, resulting in permanent protein damage [40]. Antioxidant systems serve a protective function, preventing intracellular accumulation of ROS by reversing the modification of cysteine residues [20].

The role (physiological or pathological) played by ROS depends largely on their concentration and the conditions accompanying biochemical transformations. The initial concentration dictates downstream responses [7]. Excessive amounts of ROS at the subcellular level activates pathways leading to damage in particularly susceptible cell structures or apoptosis [40]. In turn, at low physiological levels, ROS play a signalling role, essential for normal cellular processes [8,41]. Reactive oxygen species also serve as intracellular mediators produced in phagocytic cells, controlling the inflammatory response and antimicrobial defence [4].

ROS play an important role in normal thyroid function. Thyroid cells release oxidases, which catalyse ROS production [42,43,44]. Inositols are also involved in thyroid hormone synthesis and normal thyroid function, activating a cascade of processes including regulating TSH-dependent signalling (as a TSH transmitter) and generating H_2_O_2_ production used for iodination and coupling of iodotyrosine and iodothyronine [45,46,47,48]. Inositol deficiency or impairment of inositol cascades may result in insufficient synthesis of thyroid hormones, leading to hypothyroidism, which may be further compounded by an increased need for inositols in response to high TSH levels [45,48]. Myoinositol supplementation in hypothyroid patients effectively lowers TSH levels. Its effect has been demonstrated in combination with metformin and selenium compared to treatment without inositol [49,50].

**Figure 1:**
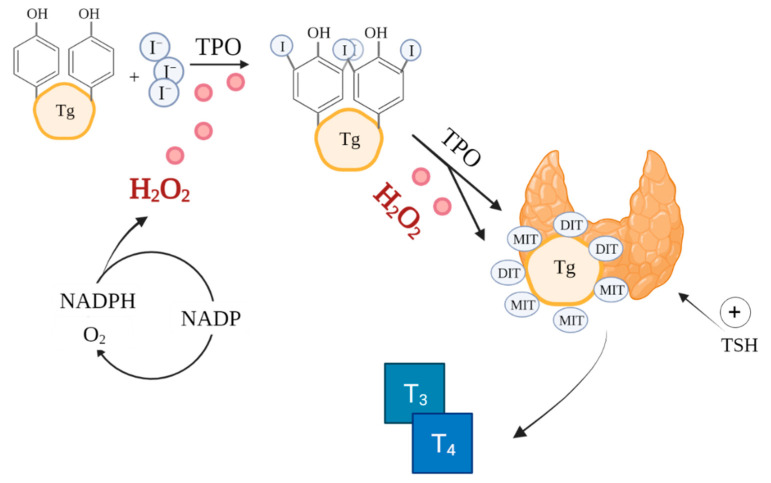
Role of ROS in thyroid hormones synthesis. Based on [47,56]. Created with BioRender.com.(accessed on 26/08/2021) I—iodine, TPO—thyroid peroxidase, Tg—thyroglobulin, MIT—monoiodotyrosine, DIT—diiodotyrosine, T_3_—triiodothyronine, T_4_—thyroxine.

The synthesis of thyroxine (T4) and triiodothyronine (T3) catalysed by thyroid peroxidase (TPO) in thyroid follicles is a very complex process involving ROS, notably, H_2_O_2_ (Figure 1) [51]. ROS are already essential in the initial stages of thyroid hormone production, during iodide oxidation [52]. Additionally, thyroid hormones perform a metabolic regulatory function by affecting mitochondrial activity [53]. Because of the reliance on ROS in its function, the thyroid is particularly exposed to oxidative damage [54]. Therefore, the antioxidant defence system of the thyroid must effectively regulate ROS production and scavenging [26,55].

## 3. Biomarkers of Oxidative Stress in Thyroid Diseases

**Figure 2:**
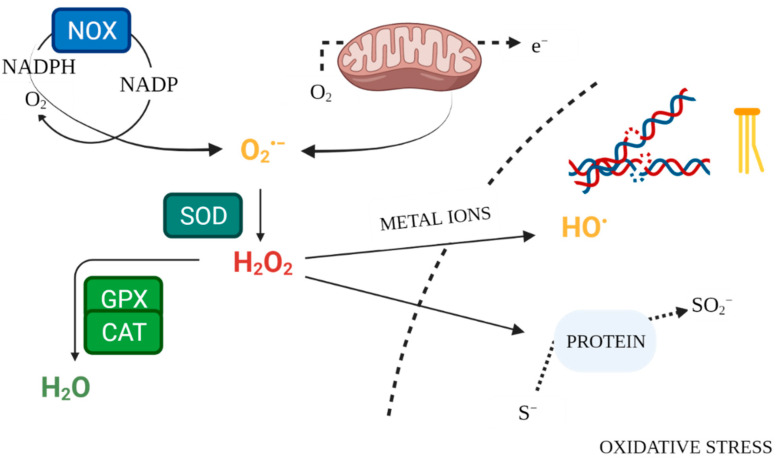
Free Radical Physiology. Created with BioRender.com. (accessed on 26 July 2021).

Enzymatic mechanisms of antioxidant defence constitute the internal system for maintaining ROS homeostasis (Figure 2). Superoxide dismutases (SOD1, SOD2, SOD3) are antioxidant enzymes, neutralising O_2_^•−^ [17,57]. The key enzyme responsible for neutralising hydrogen peroxide is catalase (CAT), which converts it to water and oxygen [58]. Likewise, glutathione peroxidase (GPX) scavenges and detoxifies H_2_O_2_ [20]. Glutathione serves as an intracellular buffer against oxidation. In response to excessive ROS release, it forms an oxidised dimer structure by bridging two glutathione molecules. Glutathione reductase (GR) then restores the reduced form of glutathione, lowering its reactivity [59]. Measurement of antioxidant enzyme activity in serum makes it possible to evaluate the condition of the antioxidant defence system. Lower levels of this activity, compared to the control, may be a sign of inadequate defence against free radicals [60].

Biomarkers of oxidative stress also include prooxidant enzymes—NADPH oxidases (NOX), which are an endogenous source of ROS, especially in thyroid tissue [46]. Their increased activity is associated with elevated concentrations of reactive oxygen species in pathological conditions. Direct measurement of ROS concentrations may be a helpful marker in the evaluation of medical conditions, yet its utility may be limited given the short half-life of these molecules [15,18].

Malondialdehyde (MDA) is a product of lipid peroxidation by ROS. The marker can be used to evaluate oxidative damage and measure whole-body or tissue-specific oxidative stress [61,62]. Advanced glycation end products (AGE) are believed to be associated with the onset and progression of metabolic disorders, notably diabetes and obesity, due to their formation both through lipid peroxidation and glycoxidation reactions; that is, in response to an increased intake of simple carbohydrates [15,63]. Elevated levels are observed in ROS-damaged tissues, as the final product of peroxidation, making them markers of oxidative stress in the body [64]. Among DNA bases, guanine is the most easily oxidised, due to its relatively low redox potential. Its oxidised form (8-oxo-2′-deoxyguanosine) may therefore serve as a measurement of DNA damage in cells exposed to oxidative stress and in carcinogenesis. 8-oxo-2′-deoxyguanosine has mutagenic potential [9,65].

Total antioxidant capacity (TAC) is a parameter indicative of the body’s overall ability to neutralise oxidants. It takes into account all the antioxidants contained in bodily fluids, including exogenous and endogenous compounds [15]. In turn, total oxidant status (TOS) is based on the oxidation of ferrous ion to ferric ion in the presence of various oxidants. It reflects the oxidation state of bodily fluids, represented by the level of radicals [66]. Oxidative stress index (OSI) is a measure of oxidative stress, calculated as the ratio of total oxidant status to total antioxidant status and therefore represents the overall oxidation state of the body [67].

All the biomarkers employed in the determination of the role of oxidative stress in thyroid diseases in this review are listed in Table 1.

## 4. Relationship between Oxidative Stress, ROS and Thyroid Diseases

### 4.1. Thyroid Disorders

#### 4.1.1. Underactive Thyroid (Hypothyroidism)

Ref [61] in hypothyroidism, including its subclinical form, elevated levels of MDA have been noted, compared to healthy individuals. Apart from inadequate antioxidant defence, this may be related to altered lipid metabolism in thyroid cells [61]. The treatment of hypothyroidism, despite lowering lipid peroxidation levels, does not bring serum MDA concentrations down to the levels observed in healthy individuals, but it may significantly boost SOD activity [73]. The relationship between hypothyroidism and oxidative stress is probably based on the lower activity of the internal antioxidant system, which does not provide adequate protection to cells against free radical accumulation, leading to oxidative damage [74]. Similarly, a mutation in the gene encoding NOX activity may contribute to excessive stimulation of ROS production. Accumulation of oxygen free radicals may inhibit TPO activity, consequently interfering with thyroid hormone production and leading to the development of hypothyroidism [46,75].

#### 4.1.2. Overactive Thyroid Gland (Hyperthyroidism)

Thyroid hormones also stimulate mitochondrial respiration, leading to an increase in ROS release in the respiratory chain. Overproduction of thyroid hormones therefore causes oxidative stress through the overproduction of free radicals, unlike in hypothyroidism, where redox imbalance can be attributed to an inefficient antioxidant defence system [74]. Consequently, overproduction of thyroid hormones (hyperthyroidism) may be associated with oxidative damage to cell structures. Individuals with hyperthyroidism present higher rates of lipid peroxidation than euthyroid individuals, which is indicative of oxidative damage to membrane lipids [76,77]. In addition, in a study investigating the effects of lead exposure on the parameters of thyroid function and antioxidant markers, thyroid hormones were shown to be positively correlated with MDA, with a positive relationship between TSH and glutathione. These findings suggest a close relationship between hyperthyroidism and the progression of oxidative stress [27].

#### 4.1.3. Thyroid Multinodules Goitre and Nodules

Elevated MDA levels were observed in tissues collected from patients with toxic and non-toxic multinodular goitre, with reduced activity of SOD, GPx and selenium content, compared to adjacent, non-pathologic tissue. Patients did not unequivocally demonstrate hyperthyroidism before surgery, as their thyroid parameters were stabilized in a euthyroid state before sampling [62]. Moreover, tissues of benign thyroid nodules show significantly reduced TAS and reduced OSI [71]. In addition, it was demonstrated that the size of thyroid nodules may decrease as a result of supplementation with extracts of plants with powerful antioxidant and anti-inflammatory properties [78]. The presence of elevated oxidative stress parameters and levels of SOD and CAT activities in toxic multinodular goitre with hyperthyroidism and decreased plasma GPx and GR activities, compared with the control group, were also demonstrated [68]. These findings suggest an impaired redox status and antioxidant defence in patients with thyroid nodules and nodular goitre.

#### 4.1.4. Autoimmune Thyroid Diseases

Chronic lymphocytic thyroiditis, also known as Hashimoto’s thyroiditis, is an autoimmune thyroid disease which presents with inflammatory cell infiltration of the thyroid gland and is characterised by the production of autoantibodies to thyroglobulin (anti-TG) and thyroperoxidase (anti-TPO) [79,80]. Inflammatory lesions in the thyroid gland result in the destruction of follicular cells and fibrosis, leading to hypothyroidism [67]. NOX participation in the production of hydrogen peroxide for the purposes of thyroid hormone synthesis may be associated with the pathophysiology of autoimmune thyroid diseases, through interacting with thyroperoxidase and thyroglobulin (TG) and altering their activity, promoting immunogenicity [75,81]. Excessive iodine intake is regarded as an additional risk factor for the development of autoimmune thyroid disease due to enhancing ROS production and, at the same time, reducing internal antioxidant levels. Anti-TPO antibodies show a dependence on glutathione levels, demonstrating an inverse relationship in individuals with Hashimoto’s thyroiditis. Additionally, both antibodies (anti-TG and anti-TPO) show a positive correlation with TOS and OSI. Decreased glutathione levels appear to be a distinctive parameter related to the activation and development of oxidative stress in Hashimoto’s thyroiditis, as oxidative stress is associated with thyroid hormone deficiency, inflammation and autoimmune parameters. Patients also present with elevated AGE levels. In addition, increased TOS and OSI parameters were shown to precede findings of hypothyroidism in autoimmune thyroiditis and could therefore be treated as predictors of thyroid cell damage [25,64,67,69,72,82].

Graves’ disease (GD) is the most common cause of hyperthyroidism and oxidative DNA damage appears to play an important role in its pathogenesis [83,84]. Enhanced inflammatory response modulates the upregulation of autoimmune response [85]. Oxidative stress, in inducing and augmenting inflammation in the thyroid, disrupts self-tolerance, consequently leading to autoimmune thyroid dysfunction. The antibodies found in GD (TSAb, thyroid stimulating antibodies) are involved in oxidation processes. The degree of DNA damage in individuals with untreated GD was shown to be significantly higher than in patients with toxic nodular goitre and individuals without thyroid dysfunction. At the same time, lipid peroxidation markers were higher than in the control. The above-mentioned parameters of oxidative stress, as well as prooxidant enzyme activity, showed a positive correlation with TSAb, suggesting their involvement in the disruption of redox homeostasis [86].

#### 4.1.5. Thyroid Cancer

**Figure 3:**
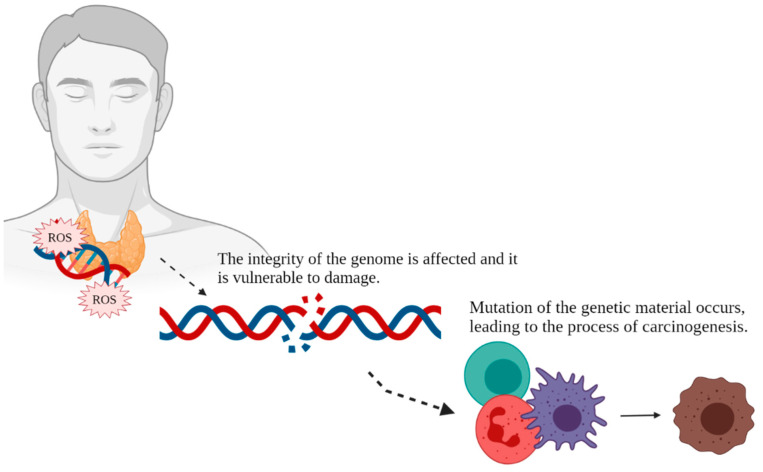
Free radical-mediated carcinogenesis in thyroid cancer. Created with BioRender.com. (accessed on 05 July 2021).

Oxidative genetic damage caused by the interaction between ROS and DNA, disrupting genomic integrity, leads to mutagenesis. Thus, oxidative stress may cause DNA damage, initiating neoplastic processes [26,87]. A simplified chart of the mechanisms of carcinogenesis, including the free-radical background, is presented in Figure 3. In murine models, oxidative damage is observed much more often in the thyroid gland than in other organs [88]. Patients with different thyroid conditions, in particular neoplasms, present higher baseline genome damage compared with healthy controls [56,89].

Patients with different types of thyroid cancer have higher serum ROS levels than healthy individuals. Apart from increased whole-body oxidation, they also present with lower activity of internal antioxidants belonging to the antioxidant defence system [60,76,89,90]. Because of the reduced activity of antioxidant enzymes in thyroid cancer cells, the inefficient defence system is not able to neutralise ROS overproduction, resulting in oxidative stress [91]. In a study evaluating the change in biomarkers of oxidative stress in individuals with thyroid cancer before and after thyroidectomy, a significant difference was demonstrated between the study and the control group in terms of glutathione peroxidase activity and MDA levels. Surgical removal of the thyroid had a significant effect on the parameters under analysis, improving oxidative status in favour of antioxidants; however, lipid peroxidation levels remained significantly higher than in healthy individuals [92]. In addition, thyroid tissues in cancer patients have altered metabolic pathways, aimed at improving cancer cell adaptation to unfavourable conditions. Metabolic pathways are shifted to promote glycolysis, more resistant to the conditions of high oxidative stress in cells. This might be an additional target for therapies aimed at processes related to cancer cell metabolism [91]. Apart from higher rates of oxidative processes in cancer patients compared to healthy individuals, those with papillary thyroid cancer had a worse oxidative profile than patients with autoimmune thyroid disease [28]. Obese patients were also found to be at an increased risk for thyroid cancer [93]. There are many reports identifying metabolic links between obesity and mitochondrial dysfunction, excessive ROS generation and oxidative stress [74,94,95,96,97]. The relationship between the development of thyroid diseases and obesity, as well as the mechanisms involved, are nevertheless unclear and require in-depth analysis and more detailed research.

## 5. Conclusions

It is most likely that many of the mechanisms participating in the development of thyroid pathologies are still unknown. However, there is a notable connection of increased ROS generation and findings of oxidative damage with the development of thyroid cancer and other diseases described here. In addition, thyroid disorders may also initiate or increase ROS release and oxidative stress, enhancing oxidative damage. The most recent studies suggest a close link between thyroid diseases and oxidative stress.

Taking into consideration research findings to date, it would appear that preventive nutrition therapy against redox imbalance, in enriching the daily diet in products with a high antioxidant value and supporting the internal antioxidant defence systems, may constitute a promising approach to preventing the development of many chronic thyroid diseases. This creates a prospect for developing measures precisely targeted at the free-radical background, which can be used in the treatment and prevention of thyroid diseases as well as other oxidative diseases.

## Figures and Tables

**Table 1:** Biomarkers of oxidative stress used in thyroid disease research [15].

Biomarkers	Mechanism of Development, Role	References
ROS	Energy metabolism in mitochondria	[68]
MDA, HNE	Lipid peroxidation products	[62]
AGE, ALE	Protein oxidation products; Advanced peroxidation end products	[64]
SOD, CAT, GPX, GR	Antioxidant enzymes	[62,68,69]
NOX, DUOX	ROS-generating enzymes	[70]
GSH/GSSG	Reduced/oxygenated glutathione	[69]
TAC, TOS	Number of moles of oxidants neutralised by one litre of body fluid; total oxidative status;	[71,72]

ROS—reactive oxygen species, MDA—malondialdehyde, HNE—hydroxynonenal, AGE-advanced glycation end products, ALE—advanced lipoxidation end products, SOD—superoxide dismutase, CAT—catalase, GPX—glutathione peroxidase, GR—glutathione reductase, NOX—NADPH oxidases, DUOX—dual oxidase, GSH/GSSG—the reduced glutathione/oxidized glutathione ratio, TAC—total antioxidant capacity, TOS—total oxidant status.

## Data Availability

Not applicable.

## References

[1] Jakubczyk K., Dec K., Kałduńska J., Kawczuga D., Kochman J., Janda K. (2020). Reactive Oxygen Species—Sources, Functions, Oxidative Damage. Pol. Merkur. Lek. Organ Pol. Tow. Lek..

[2] Tan B.L., Norhaizan M.E., Liew W.-P.-P. (2018). Nutrients and Oxidative Stress: Friend or Foe?. Oxid. Med. Cell. Longev..

[3] Yang S., Lian G. (2020). ROS and Diseases: Role in Metabolism and Energy Supply. Mol. Cell. Biochem..

[4] Shekhova E. (2020). Mitochondrial Reactive Oxygen Species as Major Effectors of Antimicrobial Immunity. PLoS Pathog..

[5] Yun H.R., Jo Y.H., Kim J., Shin Y., Kim S.S., Choi T.G. (2020). Roles of Autophagy in Oxidative Stress. Int. J. Mol. Sci..

[6] Sies H. (2015). Oxidative Stress: A Concept in Redox Biology and Medicine. Redox Biol..

[7] Di Marzo N., Chisci E., Giovannoni R. (2018). The Role of Hydrogen Peroxide in Redox-Dependent Signaling: Homeostatic and Pathological Responses in Mammalian Cells. Cells.

[8] Sies H., Berndt C., Jones D.P. (2017). Oxidative Stress. Annu. Rev. Biochem..

[9] Filomeni G., De Zio D., Cecconi F. (2015). Oxidative Stress and Autophagy: The Clash between Damage and Metabolic Needs. Cell Death Differ..

[10] Gu Y., Han J., Jiang C., Zhang Y. (2020). Biomarkers, Oxidative Stress and Autophagy in Skin Aging. Ageing Res. Rev..

[11] Vostrikova S.M., Grinev A.B., Gogvadze V.G. (2020). Reactive Oxygen Species and Antioxidants in Carcinogenesis and Tumor Therapy. Biochem. Mosc..

[12] Mahdavi A., Naeini A.A., Najafi M., Maracy M., Ghazvini M.A. (2020). Effect of Levetiracetam Drug on Antioxidant and Liver Enzymes in Epileptic Patients: Case-Control Study. Afr. Health Sci..

[13] Jakubczyk K., Kałduńska J., Dec K., Kawczuga D., Janda K. (2020). Antioxidant Properties of Small-Molecule Non-Enzymatic Compounds. Pol. Merkur. Lek. Organ Pol. Tow. Lek..

[14] Kowalska K., Brodowski J., Pokorska-Niewiada K., Szczuko M. (2020). The Change in the Content of Nutrients in Diets Eliminating Products of Animal Origin in Comparison to a Regular Diet from the Area of Middle-Eastern Europe. Nutrients.

[15] Marrocco I., Altieri F., Peluso I. (2017). Measurement and Clinical Significance of Biomarkers of Oxidative Stress in Humans. Oxid. Med. Cell. Longev..

[16] Sies H., Jones D.P. (2020). Reactive Oxygen Species (ROS) as Pleiotropic Physiological Signalling Agents. Nat. Rev. Mol. Cell Biol..

[17] Fukai T., Ushio-Fukai M. (2020). Cross-Talk between NADPH Oxidase and Mitochondria: Role in ROS Signaling and Angiogenesis. Cells.

[18] Kim Y.-M., Kim S.-J., Tatsunami R., Yamamura H., Fukai T., Ushio-Fukai M. (2017). ROS-Induced ROS Release Orchestrated by Nox4, Nox2, and Mitochondria in VEGF Signaling and Angiogenesis. Am. J. Physiol. Cell Physiol..

[19] Aldosari S., Awad M., Harrington E.O., Sellke F.W., Abid M.R. (2018). Subcellular Reactive Oxygen Species (ROS) in Cardiovascular Pathophysiology. Antioxid. Basel Switz..

[20] Irazabal M.V., Torres V.E. (2020). Reactive Oxygen Species and Redox Signaling in Chronic Kidney Disease. Cells.

[21] Garmendia Madariaga A., Santos Palacios S., Guillén-Grima F., Galofré J.C. (2014). The Incidence and Prevalence of Thyroid Dysfunction in Europe: A Meta-Analysis. J. Clin. Endocrinol. Metab..

[22] Canaris G.J., Manowitz N.R., Mayor G., Ridgway E.C. (2000). The Colorado Thyroid Disease Prevalence Study. Arch. Intern. Med..

[23] Kasagi K., Takahashi N., Inoue G., Honda T., Kawachi Y., Izumi Y. (2009). Thyroid Function in Japanese Adults as Assessed by a General Health Checkup System in Relation with Thyroid-Related Antibodies and Other Clinical Parameters. Thyroid.

[24] Empson M., Flood V., Ma G., Eastman C.J., Mitchell P. (2007). Prevalence of Thyroid Disease in an Older Australian Population. Intern. Med. J..

[25] Rostami R., Aghasi M.R., Mohammadi A., Nourooz-Zadeh J. (2013). Enhanced Oxidative Stress in Hashimoto’s Thyroiditis: Inter-Relationships to Biomarkers of Thyroid Function. Clin. Biochem..

[26] Ameziane El Hassani R., Buffet C., Leboulleux S., Dupuy C. (2019). Oxidative Stress in Thyroid Carcinomas: Biological and Clinical Significance. Endocr. Relat. Cancer.

[27] Fahim Y.A., Sharaf N.E., Hasani I.W., Ragab E.A., Abdelhakim H.K. (2020). Assessment of Thyroid Function and Oxidative Stress State in Foundry Workers Exposed to Lead. J. Health Pollut..

[28] Lassoued S., Mseddi M., Mnif F., Abid M., Guermazi F., Masmoudi H., El Feki A., Attia H. (2010). A Comparative Study of the Oxidative Profile in Graves’ Disease, Hashimoto’s Thyroiditis, and Papillary Thyroid Cancer. Biol. Trace Elem. Res..

[29] Mehran L., Amouzegar A., Rahimabad P.K., Tohidi M., Tahmasebinejad Z., Azizi F. (2017). Thyroid Function and Metabolic Syndrome: A Population-Based Thyroid Study. Horm. Metab. Res..

[30] Du F.-M., Kuang H.-Y., Duan B.-H., Liu D.-N., Yu X.-Y. (2019). Effects of Thyroid Hormone and Depression on Common Components of Central Obesity. J. Int. Med. Res..

[31] Song R.-H., Wang B., Yao Q.-M., Li Q., Jia X., Zhang J.-A. (2019). The Impact of Obesity on Thyroid Autoimmunity and Dysfunction: A Systematic Review and Meta-Analysis. Front. Immunol..

[32] Heinonen S., Buzkova J., Muniandy M., Kaksonen R., Ollikainen M., Ismail K., Hakkarainen A., Lundbom J., Lundbom N., Vuolteenaho K. (2015). Impaired Mitochondrial Biogenesis in Adipose Tissue in Acquired Obesity. Diabetes.

[33] Parra M.D., Martínez de Morentin B.E., Martínez J.A. (2005). Postprandial Insulin Response and Mitochondrial Oxidation in Obese Men Nutritionally Treated to Lose Weight. Eur. J. Clin. Nutr..

[34] Anderson E.J., Lustig M.E., Boyle K.E., Woodlief T.L., Kane D.A., Lin C.-T., Price J.W., Kang L., Rabinovitch P.S., Szeto H.H. (2009). Mitochondrial H_2_O_2_ Emission and Cellular Redox State Link Excess Fat Intake to Insulin Resistance in Both Rodents and Humans. J. Clin. Investig..

[35] Saraf-Bank S., Ahmadi A., Paknahad Z., Maracy M., Nourian M. (2019). Effects of Curcumin Supplementation on Markers of Inflammation and Oxidative Stress among Healthy Overweight and Obese Girl Adolescents: A Randomized Placebo-Controlled Clinical Trial. Phytother. Res..

[36] Yin X., Lanza I.R., Swain J.M., Sarr M.G., Nair K.S., Jensen M.D. (2014). Adipocyte Mitochondrial Function Is Reduced in Human Obesity Independent of Fat Cell Size. J. Clin. Endocrinol. Metab..

[37] Fischer B., Schöttl T., Schempp C., Fromme T., Hauner H., Klingenspor M., Skurk T. (2015). Inverse Relationship between Body Mass Index and Mitochondrial Oxidative Phosphorylation Capacity in Human Subcutaneous Adipocytes. Am. J. Physiol. Endocrinol. Metab..

[38] Christe M., Hirzel E., Lindinger A., Kern B., von Flüe M., Peterli R., Peters T., Eberle A.N., Lindinger P.W. (2013). Obesity Affects Mitochondrial Citrate Synthase in Human Omental Adipose Tissue. ISRN Obes..

[39] Schmid D., Ricci C., Behrens G., Leitzmann M.F. (2015). Adiposity and Risk of Thyroid Cancer: A Systematic Review and Meta-Analysis. Obes. Rev. Off. J. Int. Assoc. Study Obes..

[40] Schieber M., Chandel N.S. (2014). ROS Function in Redox Signaling and Oxidative Stress. Curr. Biol. CB.

[41] Sies H. (2017). Hydrogen Peroxide as a Central Redox Signaling Molecule in Physiological Oxidative Stress: Oxidative Eustress. Redox Biol..

[42] Ameziane-El-Hassani R., Schlumberger M., Dupuy C. (2016). NADPH Oxidases: New Actors in Thyroid Cancer?. Nat. Rev. Endocrinol..

[43] Cardoso L.C., Martins D.C., Figueiredo M.D., Rosenthal D., Vaisman M., Violante A.H., Carvalho D.P. (2001). Ca^2+^/Nicotinamide Adenine Dinucleotide Phosphate-Dependent H_2_O_2_ Generation Is Inhibited by Iodide in Human Thyroids. J. Clin. Endocrinol. Metab..

[44] Dupuy C., Virion A., Ohayon R., Kaniewski J., Dème D., Pommier J. (1991). Mechanism of Hydrogen Peroxide Formation Catalyzed by NADPH Oxidase in Thyroid Plasma Membrane. J. Biol. Chem..

[45] Piras C., Pibiri M., Leoni V.P., Balsamo A., Tronci L., Arisci N., Mariotti S., Atzori L. (2021). Analysis of Metabolomics Profile in Hypothyroid Patients before and after Thyroid Hormone Replacement. J. Endocrinol. Investig..

[46] Ohye H., Sugawara M. (2010). Dual Oxidase, Hydrogen Peroxide and Thyroid Diseases. Exp. Biol. Med. Maywood NJ.

[47] Benvenga S., Nordio M., Laganà A.S., Unfer V. (2021). The Role of Inositol in Thyroid Physiology and in Subclinical Hypothyroidism Management. Front. Endocrinol..

[48] Grasberger H., Van Sande J., Hag-Dahood Mahameed A., Tenenbaum-Rakover Y., Refetoff S. (2007). A Familial Thyrotropin (TSH) Receptor Mutation Provides in Vivo Evidence That the Inositol Phosphates/Ca^2+^ Cascade Mediates TSH Action on Thyroid Hormone Synthesis. J. Clin. Endocrinol. Metab..

[49] Morgante G., Musacchio M.C., Orvieto R., Massaro M.G., De Leo V. (2013). Alterations in Thyroid Function among the Different Polycystic Ovary Syndrome Phenotypes. Gynecol. Endocrinol..

[50] Pace C., Tumino D., Russo M., Le Moli R., Naselli A., Borzì G., Malandrino P., Frasca F. (2020). Role of Selenium and Myo-Inositol Supplementation on Autoimmune Thyroiditis Progression. Endocr. J..

[51] Thanas C., Ziros P.G., Chartoumpekis D.V., Renaud C.O., Sykiotis G.P. (2020). The Keap1/Nrf2 Signaling Pathway in the Thyroid—2020 Update. Antioxidants.

[52] Massart C., Hoste C., Virion A., Ruf J., Dumont J.E., Van Sande J. (2011). Cell Biology of H_2_O_2_ Generation in the Thyroid: Investigation of the Control of Dual Oxidases (DUOX) Activity in Intact Ex Vivo Thyroid Tissue and Cell Lines. Mol. Cell. Endocrinol..

[53] Venditti P., Puca A., Di Meo S. (2003). Effects of Thyroid State on H_2_O_2_ Production by Rat Heart Mitochondria: Sites of Production with Complex I- and Complex II-Linked Substrates. Horm. Metab. Res..

[54] Paunkov A., Chartoumpekis D.V., Ziros P.G., Chondrogianni N., Kensler T.W., Sykiotis G.P. (2019). Impact of Antioxidant Natural Compounds on the Thyroid Gland and Implication of the Keap1/Nrf2 Signaling Pathway. Curr. Pharm. Des..

[55] Poncin S., Gérard A.-C., Boucquey M., Senou M., Calderon P.B., Knoops B., Lengelé B., Many M.-C., Colin I.M. (2008). Oxidative Stress in the Thyroid Gland: From Harmlessness to Hazard Depending on the Iodine Content. Endocrinology.

[56] Szanto I., Pusztaszeri M., Mavromati M. (2019). H_2_O_2_ Metabolism in Normal Thyroid Cells and in Thyroid Tumorigenesis: Focus on NADPH Oxidases. Antioxidants.

[57] Eleutherio E.C.A., Magalhães R.S.S., de Araújo Brasil A., Neto J.R.M., de Holanda Paranhos L. (2021). SOD1, More than Just an Antioxidant. Arch. Biochem. Biophys..

[58] Sepasi Tehrani H., Moosavi-Movahedi A.A. (2018). Catalase and Its Mysteries. Prog. Biophys. Mol. Biol..

[59] Couto N., Wood J., Barber J. (2016). The Role of Glutathione Reductase and Related Enzymes on Cellular Redox Homoeostasis Network. Free Radic. Biol. Med..

[60] Metere A., Frezzotti F., Graves C.E., Vergine M., De Luca A., Pietraforte D., Giacomelli L. (2018). A Possible Role for Selenoprotein Glutathione Peroxidase (GPx1) and Thioredoxin Reductases (TrxR1) in Thyroid Cancer: Our Experience in Thyroid Surgery. Cancer Cell Int..

[61] Torun A.N., Kulaksizoglu S., Kulaksizoglu M., Pamuk B.O., Isbilen E., Tutuncu N.B. (2009). Serum Total Antioxidant Status and Lipid Peroxidation Marker Malondialdehyde Levels in Overt and Subclinical Hypothyroidism. Clin. Endocrinol..

[62] Erdamar H., Cimen B., Gülcemal H., Saraymen R., Yerer B., Demirci H. (2010). Increased Lipid Peroxidation and Impaired Enzymatic Antioxidant Defense Mechanism in Thyroid Tissue with Multinodular Goiter and Papillary Carcinoma. Clin. Biochem..

[63] Loomis S.J., Chen Y., Sacks D.B., Christenson E.S., Christenson R.H., Rebholz C.M., Selvin E. (2017). Cross-Sectional Analysis of AGE-CML, SRAGE, and EsRAGE with Diabetes and Cardiometabolic Risk Factors in a Community-Based Cohort. Clin. Chem..

[64] Ruggeri R.M., Giovinazzo S., Barbalace M.C., Cristani M., Alibrandi A., Vicchio T.M., Giuffrida G., Aguennouz M.H., Malaguti M., Angeloni C. (2021). Influence of Dietary Habits on Oxidative Stress Markers in Hashimoto’s Thyroiditis. Thyroid Off. J. Am. Thyroid Assoc..

[65] Kasai H. (1997). Analysis of a Form of Oxidative DNA Damage, 8-Hydroxy-2′-Deoxyguanosine, as a Marker of Cellular Oxidative Stress during Carcinogenesis. Mutat. Res. Mutat. Res..

[66] Rovcanin B.R., Gopcevic K.R., Kekic D.L., Zivaljevic V.R., Diklic A.D., Paunovic I.R. (2016). Papillary Thyroid Carcinoma: A Malignant Tumor with Increased Antioxidant Defense Capacity. Tohoku J. Exp. Med..

[67] Ates I., Arikan M.F., Altay M., Yilmaz F.M., Yilmaz N., Berker D., Guler S. (2018). The Effect of Oxidative Stress on the Progression of Hashimoto’s Thyroiditis. Arch. Physiol. Biochem..

[68] Bednarek J., Wysocki H., Sowinski J. (2004). Oxidation Products and Antioxidant Markers in Plasma of Patients with Graves’ Disease and Toxic Multinodular Goiter: Effect of Methimazole Treatment. Free Radic. Res..

[69] Rostami R., Nourooz-Zadeh S., Mohammadi A., Khalkhali H.R., Ferns G., Nourooz-Zadeh J. (2020). Serum Selenium Status and Its Interrelationship with Serum Biomarkers of Thyroid Function and Antioxidant Defense in Hashimoto’s Thyroiditis. Antioxidants.

[70] Fortunato R.S., Braga W.M.O., Ortenzi V.H., Rodrigues D.C., Andrade B.M., Miranda-Alves L., Rondinelli E., Dupuy C., Ferreira A.C.F., Carvalho D.P. (2013). Sexual Dimorphism of Thyroid Reactive Oxygen Species Production Due to Higher NADPH Oxidase 4 Expression in Female Thyroid Glands. Thyroid Off. J. Am. Thyroid Assoc..

[71] Faam B., Ghadiri A.A., Ghaffari M.A., Totonchi M., Khorsandi L. (2021). Comparing Oxidative Stress Status Among Iranian Males and Females with Malignant and Non-Malignant Thyroid Nodules. Int. J. Endocrinol. Metab..

[72] Ates I., Yilmaz F.M., Altay M., Yilmaz N., Berker D., Güler S. (2015). The Relationship between Oxidative Stress and Autoimmunity in Hashimoto’s Thyroiditis. Eur. J. Endocrinol..

[73] Baskol G., Atmaca H., Tanrıverdi F., Baskol M., Kocer D., Bayram F. (2007). Oxidative Stress and Enzymatic Antioxidant Status in Patients with Hypothyroidism before and after Treatment. Exp. Clin. Endocrinol. Diabetes.

[74] Mancini A., Di Segni C., Raimondo S., Olivieri G., Silvestrini A., Meucci E., Currò D. (2016). Thyroid Hormones, Oxidative Stress, and Inflammation. Mediators Inflamm..

[75] Fortunato R.S., Ferreira A.C.F., Hecht F., Dupuy C., Carvalho D.P. (2014). Sexual Dimorphism and Thyroid Dysfunction: A Matter of Oxidative Stress?. J. Endocrinol..

[76] Wang D., Feng J.-F., Zeng P., Yang Y.-H., Luo J., Yang Y.-W. (2011). Total Oxidant/Antioxidant Status in Sera of Patients with Thyroid Cancers. Endocr. Relat. Cancer.

[77] Piazera B.K.L., Gomes D.V., Vigário P., Salerno V.P., Vaisman M. (2018). Evaluation of Redox Profiles in Exogenous Subclinical Hyperthyroidism at Two Different Levels of TSH Suppression. Arch. Endocrinol. Metab..

[78] Stancioiu F., Mihai D., Papadakis G.Z., Tsatsakis A., Spandidos D.A., Badiu C. (2019). Treatment for Benign Thyroid Nodules with a Combination of Natural Extracts. Mol. Med. Rep..

[79] Burek C.L., Rose N.R. (2008). Autoimmune Thyroiditis and ROS. Autoimmun. Rev..

[80] Laganà A.S., Santoro G., Triolo O., Giacobbe V., Certo R., Palmara V. (2015). Hashimoto Thyroiditis Onset after Laparoscopic Removal of Struma Ovarii: An Overview to Unravel a Rare and Intriguing Finding. Clin. Exp. Obstet. Gynecol..

[81] Duthoit C., Estienne V., Giraud A., Durand-Gorde J.M., Rasmussen A.K., Feldt-Rasmussen U., Carayon P., Ruf J. (2001). Hydrogen Peroxide-Induced Production of a 40 KDa Immunoreactive Thyroglobulin Fragment in Human Thyroid Cells: The Onset of Thyroid Autoimmunity?. Biochem. J..

[82] Baser H., Can U., Baser S., Yerlikaya F.H., Aslan U., Hidayetoglu B.T. (2015). Assesment of Oxidative Status and Its Association with Thyroid Autoantibodies in Patients with Euthyroid Autoimmune Thyroiditis. Endocrine.

[83] Zarković M. (2012). The Role of Oxidative Stress on the Pathogenesis of Graves’ Disease. J. Thyroid Res..

[84] De Leo S., Lee S.Y., Braverman L.E. (2016). Hyperthyroidism. Lancet Lond. Engl..

[85] Rasool M., Malik A., Saleem S., Ashraf M.A.B., Khan A.Q., Waquar S., Zahid A., Shaheen S., Abu-Elmagd M., Gauthaman K. (2021). Role of Oxidative Stress and the Identification of Biomarkers Associated With Thyroid Dysfunction in Schizophrenics. Front. Pharmacol..

[86] Diana T., Daiber A., Oelze M., Neumann S., Olivo P.D., Kanitz M., Stamm P., Kahaly G.J. (2018). Stimulatory TSH-Receptor Antibodies and Oxidative Stress in Graves Disease. J. Clin. Endocrinol. Metab..

[87] Nakashima M., Suzuki K., Meirmanov S., Naruke Y., Matsuu-Matsuyama M., Shichijo K., Saenko V., Kondo H., Hayashi T., Ito M. (2008). Foci Formation of P53-Binding Protein 1 in Thyroid Tumors: Activation of Genomic Instability during Thyroid Carcinogenesis. Int. J. Cancer.

[88] Maier J., van Steeg H., van Oostrom C., Karger S., Paschke R., Krohn K. (2006). Deoxyribonucleic Acid Damage and Spontaneous Mutagenesis in the Thyroid Gland of Rats and Mice. Endocrinology.

[89] Gerić M., Domijan A.M., Gluščić V., Janušić R., Šarčević B., Garaj-Vrhovac V. (2016). Cytogenetic Status and Oxidative Stress Parameters in Patients with Thyroid Diseases. Mutat. Res. Toxicol. Environ. Mutagen..

[90] Ramli N.S.F., Mat Junit S., Leong N.K., Razali N., Jayapalan J.J., Abdul Aziz A. (2017). Analyses of Antioxidant Status and Nucleotide Alterations in Genes Encoding Antioxidant Enzymes in Patients with Benign and Malignant Thyroid Disorders. PeerJ.

[91] Metere A., Graves C.E., Chirico M., Caramujo M.J., Pisanu M.E., Iorio E. (2020). Metabolomic Reprogramming Detected by 1H-NMR Spectroscopy in Human Thyroid Cancer Tissues. Biology.

[92] Akinci M., Kosova F., Çetin B., Sepici A., Altan N., Aslan S., Çetin A. (2008). Oxidant/Antioxidant Balance in Patients with Thyroid Cancer. Acta Cirúrgica Bras..

[93] Oberman B., Khaku A., Camacho F., Goldenberg D. (2015). Relationship between Obesity, Diabetes and the Risk of Thyroid Cancer. Am. J. Otolaryngol..

[94] Kanikowska D., Kanikowska A., Swora-Cwynar E., Grzymisławski M., Sato M., Bręborowicz A., Witowski J., Korybalska K. (2021). Moderate Caloric Restriction Partially Improved Oxidative Stress Markers in Obese Humans. Antioxidants.

[95] Włodarczyk M., Nowicka G. (2019). Obesity, DNA Damage, and Development of Obesity-Related Diseases. Int. J. Mol. Sci..

[96] Lahera V., de Las Heras N., López-Farré A., Manucha W., Ferder L. (2017). Role of Mitochondrial Dysfunction in Hypertension and Obesity. Curr. Hypertens. Rep..

[97] Zaki M., Basha W., El-Bassyouni H.T., El-Toukhy S., Hussein T. (2018). Evaluation of DNA Damage Profile in Obese Women and Its Association to Risk of Metabolic Syndrome, Polycystic Ovary Syndrome and Recurrent Preeclampsia. Genes Dis..

